# Expansion microscopy: A chemical approach for super-resolution microscopy

**DOI:** 10.1016/j.sbi.2023.102614

**Published:** 2023-05-28

**Authors:** Yinyin Zhuang, Xiaoyu Shi

**Affiliations:** 1 Department of Developmental and Cell Biology, University of California, Irvine, CA 92697, USA; 2 Department of Chemistry, University of California, Irvine, CA 92697, USA; 3 Department of Biomedical Engineering, University of California, Irvine, CA 92697, USA

## Abstract

Super-resolution microscopy is a series of imaging techniques that bypass the diffraction limit of resolution. Since the 1990s, optical approaches, such as single-molecular localization microscopy, have allowed us to visualize biological samples from the sub-organelle to the molecular level. Recently, a chemical approach called expansion microscopy emerged as a new trend in super-resolution microscopy. It physically enlarges cells and tissues, which leads to an increase in the effective resolution of any microscope by the length expansion factor. Compared with optical approaches, expansion microscopy has a lower cost and higher imaging depth but requires a more complex procedure. The integration of expansion microscopy and advanced microscopes significantly pushed forward the boundary of super-resolution microscopy. This review covers the current state of the art in expansion microscopy, including the latest methods and their applications, as well as challenges and opportunities for future research.

## Introduction

Since the 1990s, optical approaches to bypass the diffraction limit have revolutionized how we visualize organelles and sub-organelle structures, such as nuclear pore complexes (NPCs) and centrioles, which could not be dissected by conventional light microscopy. These super-resolution approaches rely on advanced microscopes, such as structured illumination microscopy (SIM) [1], single-molecule localization microscopy [2–4], and stimulated emission depletion microscopy (STED) [5]. These optical approaches achieved super-resolution ranging from 7 to 150 nm [6,7]. In 2014, the Nobel prize in chemistry was awarded to Stefan Hell, Eric Betzig, and William Moerner for their contribution to super-resolution light microscopy. However, biophysicists’ creativity and passion for super-resolution imaging are far from ending. This review focuses on a new trend in super-resolution microscopy (SRM), which is a chemical approach called expansion microscopy (ExM).

Distinct from optical approaches, ExM methods physically enlarge cells and tissues in swellable hydrogel through chemical procedures. As a result, the effective resolution of any microscope, including conventional light microscopes and SRMs, is increased by the expansion factor of the sample. Edward Boyden’s lab first applied this sample enlargement concept in super-resolution imaging and named it ExM [8]. Over the past few years, various ExM techniques have been developed for different biomolecules and biological systems. Its combination with SRMs significantly narrows the gap between light and electron microscopy.

Compared with optical super-resolution approaches, the most significant advantages of ExM are its low cost, high imaging depth, and tissue clearing. However, there are still challenges to solve, standards to set, and applications to explore. This review will discuss the principles, recent advances, applications, challenges and solutions, and future directions of ExM. We intend to guide biologists in selecting suitable ExM techniques and SRM combinations for different purposes in biological research.

### Principles of ExM

#### Principle and workflow

**Figure 1:**
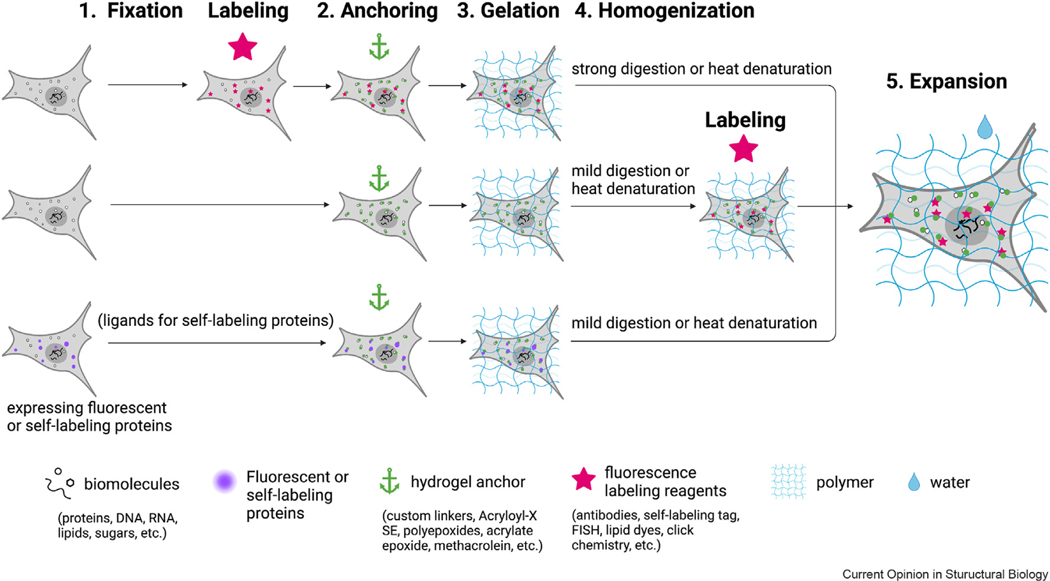
Three common workflows for expansion microscopy. The first is the original expansion pathway, where samples are fluorescently labeled first and enlarged through anchoring, gelation, homogenization, and expansion steps (top). Alternatively, target biomolecules can be fluorescently labeled after anchoring, gelation, and homogenization (middle). For samples expressing fluorescent proteins, special anchoring crosslinkers and mild homogenization methods can be used to retain the fluorescence signal for imaging after expansion (bottom). Finally, for samples expressing self-labeling protein tags, labeling with ligands that recognize the protein tags is required before anchoring (bottom).

All the expansion techniques have a common goal: isotropically expanding biological samples in three dimensions [9]. To achieve this goal, five steps are required in the workflow of most ExM methods (Figure 1): First, fix and permeabilize the sample. Second, modify the sample with chemical anchors. Third, form *in situ* swellable hydrogel in the sample, where biomolecules are crosslinked to the hydrogel by the chemical anchors. Fourth, homogenize the gel sample via enzyme digestion or heat denaturation to break interactions between biomolecules. Fifth, expand the gel sample with water. The electrolytes in swellable hydrogel make the gel absorb water and expand. Fluorescence labeling of targeted biomolecules can be done before the hydrogel forms, such as in ExM [8], or after the gelation, such as in magnified analysis of the proteome (MAP) [10]. Each order has its advantages and disadvantages, which will be discussed in Challenges in expansion microscopy and solutions, Challenges in ExM and solutions.

The effective resolution of ExM $\left(d_{eff}\right)$ is defined by two elements: the resolution of the microscope $\left(d_{micro}\right)$ and the length expansion factor of the hydrogel $\left(\varepsilon_{expan}\right)$.


[Equation 1] $$d_{eff}=\frac{d_{micro}}{\varepsilon_{expan}}$$

For example, if a 250 nm-resolution epifluorescence microscope is used to image cells expanded by 4.0 times in each dimension, the effective resolution would be 62.5 nm. Imaging on SRMs or expanding samples with larger expansion factors can further increase the effective resolution.

#### Expansion factor and the resolution limit

A higher expansion factor $\varepsilon_{expan}$ means higher resolution in ExM. The expansion factor is determined by the gel osmotic pressure arising from the polymer and biomolecule concentrations, gel-sample hybrid system elasticity, and mobile ions in the hybrid system [11]. The basic gel formula consists of: first, a monomer base that is a mix of uncharged acrylamide and ionic acrylate for generating polymer chains; second, crosslinkers like $\text{N}-{\text{N}}^{\prime }$ -methylenebisacrylamide for connecting the polymer chains; third, initiator such as ammonium persulfate for triggering the free-radical polymerization; fourth, tetramethylethylenediamine for accelerating the reaction. Increasing the concentration of monomers is a common strategy to maximize the expansion factor, such as x10 ExM protocol [12], which expands the sample 10 times. Lowering the concentration of crosslinkers, which increases gel elasticity, also elevates the expansion factor. It is demonstrated in the ten-fold robust expansion microscopy (TREx) [13], which expands samples from 4 to 14 times. Another strategy is the long-time hydrolysis of polyacrylamide, which makes the gel expand more by increasing its ionic residues [11]. A method called Zoom by hydrogel conversion microscopy utilized this strategy and achieved an 8-fold increase through the inclusion of a 24-h heating step in the standard expansion process [11]. Furthermore, iterative expansion (iExM) [14,15] expanded samples by more than 20 times, via multiple cycles of gelation and expansion.

The highly expanded gel can be combined with SRM approaches for even higher resolution. However, there is a limitation to the maximum effective resolution of ExM. Technically, the ultimate resolution is constrained by the pore size of the hydrogel before expansion. Because the pore size determines how fine the hydrogel can faithfully anchor the biomolecules in their initial positions. Any structural details smaller than the pore size are distorted.

#### Achieving molecular resolution by combining with SRM

**Figure 2:**
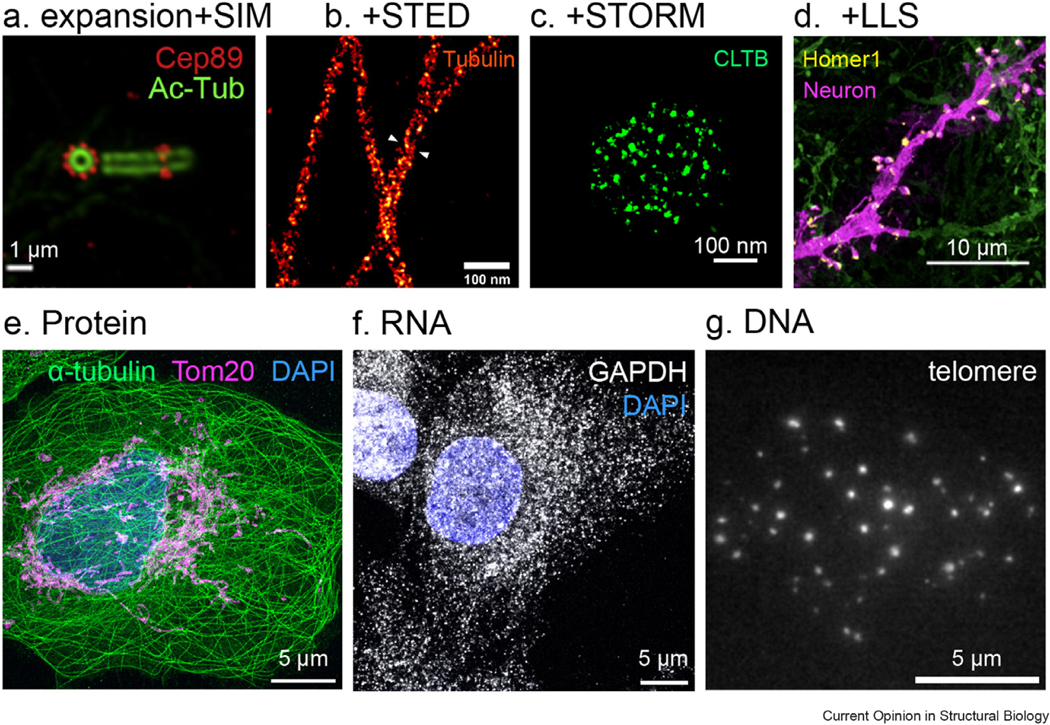
Expansion microscopy images of proteins, RNAs and DNAs on different types of microscopes. **(a)** U-ExM structured illumination microscopy (SIM) image of cilia immunostained with Cep89 (red) and acetylated tubulin (green) [24]. **(b)** ExSTED image of microtubules stained with tubulin [25]. **(c)** LR-ExSTORM image of clathrin-coated pits in a HeLa cell overexpressing SNAP-clathrin light chain b (CLTB) [19]. **(d)** Expansion lattice light-sheet (ExLLS) image of neuron (magenta, green) immunostained with synaptic proteins Homer1 (yellow) [50]. **(e)** LR-ExM confocal image of microtubules labeled with anti-α-tubulin antibodies (green), mitochondria labeled with anti-Tom20 antibodies (magenta), and DNA stained with DAPI (4′,6-diamidino-2-phenylindole) (blue) in a U2OS cell (data from our lab). **(f)** ExFISH confocal image of GAPDH labeled with DNA oligos (gray) and DNA (blue) of a HeLa cell (data from our lab). **(g)** ExM image of telomere (gray) in chromatin of an IMCD3 cell (data from our lab). Scale bars: 100 nm (a and c), 1 μm (b), 10 μm (d), 5 μm (e–g).

Combining ExM with optical SRM will allow molecular resolution imaging. However, the implementation of the combination is more complex. Several technical challenges must be overcome. First, since the sample is expanded 4 times in axial and lateral directions, the microscope needs to provide higher imaging depth for whole-cell imaging. Second, a mounting medium containing thiols and oxygen scavengers is required to switch on and off the fluorophores in many SRM techniques, such as stochastic optical reconstruction microscopy (STORM) and photoactivated localization microscopy [4,16]. The osmotic pressure induced by the mounting medium causes gel shrinking. Third, the loss of fluorophore signals during the expansion process poses a major obstacle to meeting the high standard for labeling density in molecular resolution imaging [17]. In the past seven years, many new expansion protocols have been developed to overcome these barriers. For example, water-immersion objectives offer a higher imaging depth for ExM. Re-embedding the expanded samples into an uncharged polyacrylamide gel reduced the shrinking of hydrogel in SRM mounting medium [18]. The employment of self-labeling small tag probes, such as Snap-tag [19], organic fluorescent probe, such as semiconducting polymer dots [20] or amplified immunostaining [21], significantly enhances labeling efficiency. These approaches paved the way for the combination of ExM and SRM (Figure 2a-d). Expansion SIM provided an effective lateral resolution of 30 nm (Figure 2a) [22–24], while expansion STED achieved 10 nm lateral and 50 nm axial resolution (Figure 2b) [25,26]. Label-retention expansion STORM resolved SNAP-tag labeled clathrin lattice at a final resolution of 5 nm (Figure 2c) [19]. Recently, combining ExM with fluorescence fluctuation-based SRM, such as super-resolution optical fluctuation imaging [20] and super-resolution radial fluctuations (SRRF) [27,28], achieved resolution at 25 nm. Different combinations of ExM and SRM techniques result in a range of effective resolution and imaging depth. Table 1 provides an overview of the distinct characteristics of various combinations to assist readers in selecting the appropriate imaging approach.

### Recent advances in ExM

#### Visualizing specific biomolecules

Various expansion techniques are developed to image proteins, nucleic acids, lipids, and sugars. Early versions of expansion methods, such as proExM and MAP, focused on protein imaging (Figure 2e) [8,10,17,29]. Later, expansion methods extended to RNAs (Figure 2f) and DNAs (Figure 2g), such as expansion fluorescent *in situ* hybridization (ExFISH) [30] and Single Cell Evaluation of Post-TRanslational Epigenetic Encoding [31]. Recently, Click-ExM developed by Xing Chen lab enables expansion imaging of various biomolecules, including lipids, glycans, DNAs, and metabolites [32]. A notable trend in ExM is to image the landscape of all proteins, lipids, or carbohydrates in cells and tissues at molecular resolution. Electron-microscopy-like images were obtained by these methods, such as pan-ExM [15], fluorescent labeling of abundant reactive entities (FLARE) [33], membrane ExM [34], lipid ExM [35], and click-ExM [32]. Technically, ExM can image any biomolecules if there is a method to label the molecules of interest, a way to anchor the molecules or labels to the hydrogel and to digest the molecular scaffold.

Different types of biomolecules require specific labeling methods. Immunofluorescence is the most common labeling method for ExM imaging of targeted proteins. Signals from fluorescent proteins can also be imaged with expansion protocols using special crosslinkers, such as polyepoxides [36], or mild denaturation [29]. Label-retention expansion microscopy (LR-ExM) [19] and TRIvalenT anchOriNg (TRITON) [37] introduced multifunctional chemical labels for antibody-free targeting of proteins. Nevertheless, protein probes are not limited to fluorophores. A recent method called unclearing microscopy developed by Joerg et al. uses visible chromogenic probes to stain proteins, with which cells can be seen with the naked eyes after 20 times expansion [38]. To label RNA and DNA loci with ExM, FISH is widely used [30,31]. For lipid imaging, small lipophilic molecules such as membrane-binding fluorophore-cysteine-lysine-palmtoyl group (mCling), 1,2-distearoyl-sn-glycero-3-phosphoethanolamine, and 1,1′-dioctadecyl-3,3,3′,3′-tetramethylindodicarbocyanine, 4-chlorobenzenesulfonate salt (DiD) are employed [13,34,37,39]. Recently, metabolic labeling was adopted as a powerful tool in the expansion imaging of newly synthesized nucleic acids, lipids, and glycans. For instance, Click-ExM used 5-ethynyl-2′-deoxyuridine labeling for DNA, alkyne-choline for lipids, and azido sialic acid for glycan [32].

To retain the labels in the hydrogel, anchoring reagents are applied after labeling in most expansion protocols. The anchoring reagents are crosslinkers that covalently connect target biomolecules with the hydrogel. For example, methacrylic N-hydroxysuccinimide (NHS) ester reacts with primary amines of proteins with the NHS ester group and inserts its methacrylamide group into the polyacrylic chains of hydrogel [29]. ExFISH uses an anchor that contains an alkylating group for labeling guanine of RNA and an acrylamide group to crosslink to the hydrogel [30]. Most recently, researchers developed universal anchoring strategies for different biomolecules, potentially making ExM more versatile [39,40]. For instance, Edward Boyden’s lab uses acrylate epoxides in the unified expansion microscopy (uniExM) [40], while Yongxin Zhao et al. use methacrolein in Magnify [39]. Both methods anchor multiple types of biomolecules, such as nucleic acids, proteins, and lipids, with one reagent.

#### Applications in biological systems

ExM methods have been disseminated to almost every biological system, which includes but are not limited to cell lines [8,19], bacteria [41–43], virus [44,45], yeast [46], c elegans [47], zebrafish [48], drosophila [24], tissues [13,49–51], organoids [52,53], organs [10,54], and plants [55,56]. Each biological system has a unique structure and requires a specialized expansion protocol. The significant differences among these protocols are in the denaturation or digestion step. For example, the *C. elegans* expansion protocols have an additional collagenase digestion step, which permeates the cuticles of c elegans. Bacteria expansion protocols require adaption of digestion strategy to the change of cell wall conditions among cell type and cell cycle phases. The cited references above extensively discussed specific requirements for individual biological systems.

ExM has shown potential in various applications for human samples, such as neuroscience, cancer research, and pathology. It enables nanoscale imaging of neural circuits and their connections, providing valuable in-sights into neurological diseases and disorders [57,58]. ExM has also been used to investigate cancer cells’ interactions with surrounding cells, the extracellular matrix, and blood vessels [59]. This information helps in developing better-targeted therapies and understanding tumor progression. One of the applications in pathology is expansion pathology, where the technique is used for diagnostic purposes [51]. When imaging expanded tissue samples, scientists can achieve higher accuracy in identifying diseased tissue, leading to advancements in diagnostics and research for various medical conditions.

The most straightforward expansion protocols are for cultured cells. Organelles, such as microtubules, centrioles, cilia, mitochondria, and NPCs, are used to benchmark new expansion protocols [13,15,19,25,26,60]. Beginners can start with cultured cells to practice the lengthy expansion procedures. Among the applications of ExM in biology and medicine, those in neuroscience matured the fastest, which have been nicely reviewed by Gallagher and Zhao [57]. The least known is ExM for plant science. However, it has been catching up recently. Kao and Nodine [55] and Kubalova et al. [56] reported successful expansion imaging of Arabidopsis seeds and barley nuclei, respectively.

### Challenges in ExM and solutions

We must overcome two significant challenges in signal retention and isotropic expansion to make ExM an off-the-shelf super-resolution approach. The following subsections discuss the causes and solutions to fluorescence loss and anisotropic expansion.

#### Fluorescence signal loss

Fluorescence signal loss after the expansion has been limiting the dissemination of ExM. On average, about 50% of fluorescence can be missing in early versions of expansion protocols [17,61]. Gelation and sample homogenization steps in expansion procedure are the major causes of fluorescence loss. First, the hydrogel is formed through free radical chain reactions, which destroy fluorophores or permanently lock them in a dark state [8,17,61]. Some fluorescent dyes, such as Alexa Fluor 488 and 568, better survive the radical bombardment than others. However, many photoswitchable dyes for SRM, such as Alexa Fluor 647 and Cy5, suffer more than 90% loss after the polymerization [17,29,62]. Second, fluorophores that are not anchored to the hydrogel are washed out after the tissue homogenization step, primarily when proteinase digestion is used. The fluorescent dyes are not covalently anchored to the hydrogel in many expansion methods. These dyes stay in place because the antibodies they conjugate to are crosslinked to the hydrogel. However, over-digestion fragments proteins, including antibodies. Incompletely anchored anti-body fragments and the fluorescent dye on them will be washed out after the over-digestion [29,63,64].

There are several strategies to compensate for the fluorescence loss during expansion, including signal amplification, post-gelation immunostaining, and staining with label-retention probes. Amplifying remaining labels using immunosignal hybridization chain reaction [21], primer exchange reaction [65], or biotin [29,62] can significantly enhance the fluorescent signal. These methods work well with confocal and light-sheet microscopes. However, signal amplification cannot recover the lost positional information from washed-out antibodies, resulting in brightly but incompletely labeled structures. The incomplete labeling will be detected under SRMs, such as STORM, STED, and SIM. A solution to prevent fluorescence loss from being washed out is post-gelation immunostaining. For example, MAP [10], stabilization under harsh conditions via intra-molecular epoxide linkages to prevent degradation [36], and clear unobstructed brain/body imaging cocktails and computational analysis-X [54] introduce immunofluorescence after polymerization and homogenization. This approach avoided fluorescence loss and enabled better accessibility of antibodies. Yet, the homogenization step can still damage certain epitopes, which antibodies cannot recognize after gelation. Ku et al. evaluated a comprehensive list of proteins using MAP and suggested antibodies compatible with the post-gelation immunostaining [10].

Recently, label-retention multifunctional probes emerged as a new strategy to prevent fluorescence loss completely. The multifunctional probes are small molecules that consist of at least three functional groups, a connector to recognize the target biomolecules, an anchor to crosslink to hydrogel, and a reporter that can be fluorescently labeled after expansion for imaging. Because these probes covalently anchor themselves to hydrogel, signals remain where targeted molecules, even if all targeted molecules are digested and washed out. The trifunctional probes that our lab developed for LR-ExM have an NHS ester group to conjugate antibodies, a methacrylamide group to insert into the polyacrylic hydrogel, and a biotin or digoxigenin group that allows fluorescence labeling after homogenization [19]. The NHS ester can also be replaced with ligands for SNAP and CLIP tags, which are particularly advantageous because of their high labeling efficiency and small size [19]. Johan Hofken et al. designed TRITON trivalent probes, which enabled antibody-free targeting of actin, phospholipid, and RNAs [37,66]. The multifunctional probes work well with most SRMs, such as STORM and STED, providing unprecedented resolution. However, the limited synthesis scale of label-retention probes in research labs hampers the widespread adoption of these new techniques. Thankfully, there are funding mechanisms in place to promote the spread of emerging imaging technologies. Our lab is leveraging such funding resources to scale up the synthesis of our label-retention probes and distribute them to the scientific community before they are commercialized. See funding details in the Acknowledgments section.

#### Anisotropic expansion

Isotropic expansion is the key to ExM’s success because expanded samples must faithfully preserve spatial relationships among biomolecules. However, anisotropic expansion happens in improper protocols, which causes structural distortion. Distortion typically occurs in protein-dense complexes, such as NPCs and centrioles, which are expanded less than other organelles. But many research groups have successfully expanded these structures with no problems [26]. Based on their methods and our own experience, we identified three main causes of the structure distortion: over-crosslinking of proteins, incomplete sample homogenization, and low mechanical stability of the hydrogel.

Over-crosslinking of proteins during fixation is a common reason for anisotropic expansion. Choosing the proper fixation method can significantly reduce the local distortion. Ultrastructure expansion microscopy (U-ExM) achieved near-native structural expansion of cellular contents using mild fixation followed by incubation of samples in a combination of low formaldehyde and acrylamide [26]. Based on the U-ExM, cryofixation ExM further preserves the native structural organization of cellular contents via rapid cryo-fixation [67].

**Figure 3:**
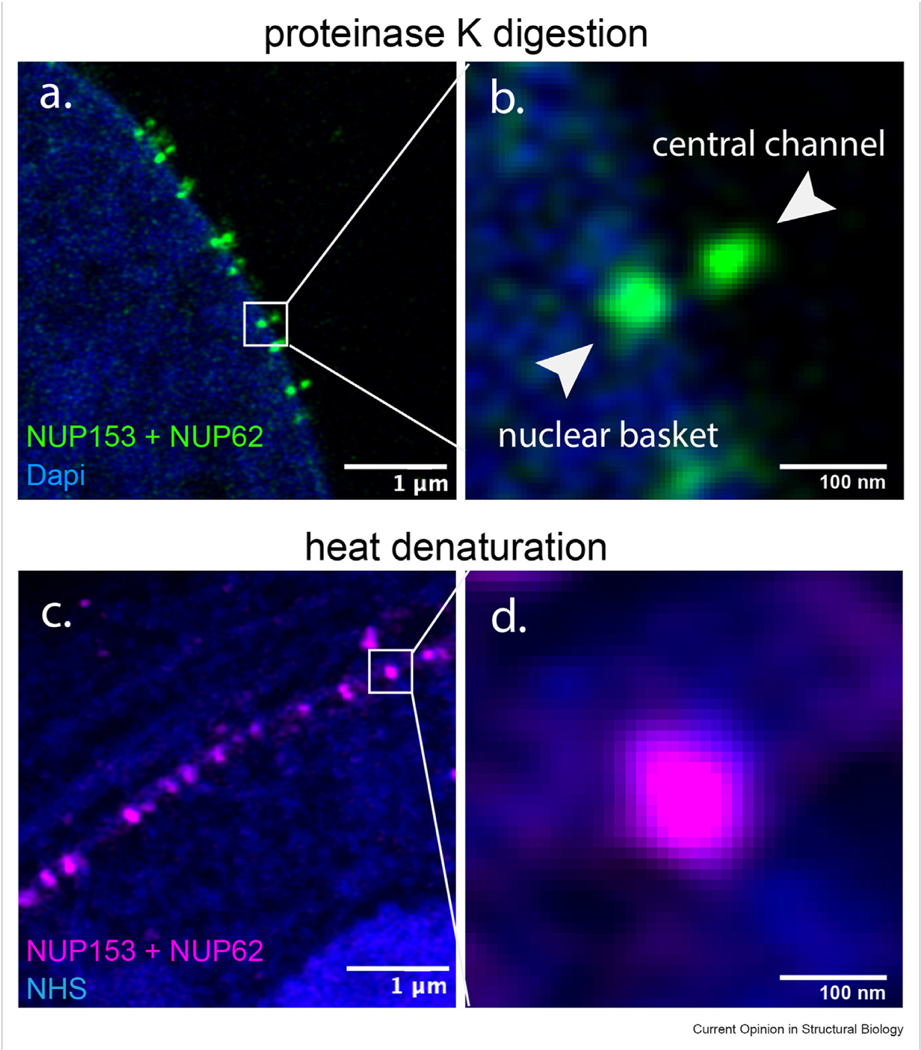
Incomplete homogenization results in anisotropic expansion of NPCs (data from our lab). All panels are LR-ExM of nuclear pore complexes (green in a and b, magenta in c and d) labeled with anti-NUP62 and anti-NUP153 antibodies taken with an Airyscan microscope. Complete homogenization using proteinase K digestion results in well-expanded NPCs (**a**), where the central channel and nuclear basket of each NPC are distinguishable in the magnified view (**b**) of the white block in panel (a). Incomplete homogenization using heat denaturation results in less expanded NPCs (**c**), where the central channel and nuclear basket are indistinguishable in the magnified view (**d**) of the white block in panel (c). The blue channel in (a and b) is DNA stained with DAPI. And the blue channel in (c and d) is whole protein staining with NHS ester dyes. Scale bars: 1 μm (a and c), 100 nm (b and d).

Incomplete sample homogenization is another major cause of structural distortion. Compared with heat denaturation, proteolysis offers more efficient sample homogenization. As depicted in Figure 3 (data from our lab), the central channel and nuclear basket of the NPCs were separated when homogenized using proteinase K digestion (Figures 3a and b), but indistinguishable through heat denaturation (Figures 3c and d). However, the drawback of using proteinase K is that it causes severe signal loss. Solutions to this drawback can be found in Fluorescence signal loss.

Using new types of harder-to-deform hydrogels can also make expansion more uniform across the sample. A highly homogeneous polymer composed of tetrahedron-like monomers was reported for high-isotropy ExM [68]. In summary, to achieve isotropic expansion, it is necessary to perform systematic optimization of fixation, homogenization, and mechanical stability of the hydrogel for each specific biological system.

### Conclusion and guidance for method choosing

In this review, we demystified ExM as a chemical approach for super-resolution imaging and showcased its great potential in widespread applications in biology. We have discussed the working principles of ExM, recent advances in this technology, applications in various biological systems, and the challenges and solutions for ExM. Compared with the optical approaches, ExM has a lower cost, lower requirement for microscopes, and higher imaging depth. The combination of ExM and optical SRM pushes forward the resolution of light microscopy to the molecular level. ExM has been adapted and applied to various biological systems, such as cell lines [8,19], bacteria [41–43], virus [44,45], yeast [46], c elegans [47], zebrafish [48], drosophila [24], tissues [13,49–51], organoids [52,53], organs [10,54], and plants [55,56] as discussed in Applications in biological systems. The successful applications of ExM on tissues and organs enable large-scale multi-omic investigation with nanoscale precision. Since the ExM protocols are specialized, the users should choose their optimal protocol and imaging system according to the target resolution (Table 1, Principles of expansion microscopy), target molecules Visualizing specific biomolecules, biological models Applications in biological systems, signal intensity Fluorescence signal loss, and protein density of organelles Anisotropic expansion. We hope these sections will guide biologists to identify the matching expansion technologies to their biological questions.

### Future directions

Undoubtedly, it is an exciting time for both method development and applications of ExM. ExM and its combination with optical SRM have narrowed the gap between light and electron microscopy. The whole-protein and whole-lipid ExM, such as pan-ExM and FLARE, produced electron-microscopy-comparable images. It allows the visualization of targeted proteins in their protein and lipid context with the molecular resolution [15,18,26,67]. The combination of ExM with SRRF (ONE) further enables ExM’s resolution to 1 nm [28]. These methods uncover similar information as correlative light and electron microscopy does but with lower cost and faster turnaround. However, we still do not have a robust readout to evaluate the local distortion of expanded samples. A method called GelMap recently reported expansion isotropy by introducing a fluorescent grid together with cultured cells into the hydrogel [69]. However, the fluorescent grid is only located on the 2D basal plane of samples. Methods that can map gel expansion homogeneity in 3D are still urgently needed because biomolecules are arranged in 3D cellular architecture.

As expansion technologies mature, applications emerge in new areas beyond cells and animal models. A recent application studied the cell-material interface [70]. Via expanding cells cultured on nanopillars, Biaoxiao Cui Lab visualized the sub-diffraction-limited interface between cells and these nanoscale topologies, which could only be imaged by electron microscopy previously. In the near future, we look forward to more ground-breaking applications in organelle–organelle interactions, cell-material interplay, structure–function relationships, neuroscience, host–microbiota interactions, and disease mechanisms.

## Figures and Tables

**Table 1:** Comparison of combinations of Expansion Microscopy with different SRM methods.

	Effective lateral resolution^b^ $d_{eff}$	Maximum Effective imaging depth^c^	Photodamage	Probe
x4 expansion^a^ +Epifluorescence/Confocal	~70 nm	15 μm	Medium	Conventional fluorophores
x4 expansion + Light Sheet/Lattice Light Sheet	~70 nm	15 μm	Low	Conventional fluorophores
x4 expansion + SIM	25–40 nm	<4 μm	Medium	Conventional fluorophores
x4 expansion + Airyscan	30–40 nm	15 μm	Medium	Conventional fluorophores
x4 expansion + SOFI/SRRF	15–30 nm	<2 μm higher when using light sheet	Medium	Fluctuating fluorophores
x4 expansion + STED	~10 nm	15 μm	High	High-depletability fluorophores
x4 expansion + PALM/STORM/SMLM	5–10 nm	<2 μm higher when using light sheet	Medium	Photoactivatable fluorophores

a The x4 expansion refers to samples that have been expanded by a factor of 4.0. The effective resolution is calculated with a 4.0 length expansion factor (Equation (1)).

b The maximum effective lateral resolution is calculated as the maximum lateral resolution of the microscope divided by the length expansion factor of the gel sample.

c The maximum effective imaging depth is the maximum imaging depth of the microscope divided by the length expansion factor.

## Data Availability

Data will be made available on request.
